# Antagonizing peroxisome proliferator‐activated receptor γ facilitates M1‐to‐M2 shift of microglia by enhancing autophagy via the LKB1–AMPK signaling pathway

**DOI:** 10.1111/acel.12774

**Published:** 2018-05-08

**Authors:** Juan Ji, Teng‐Fei Xue, Xu‐Dong Guo, Jin Yang, Ruo‐Bing Guo, Juan Wang, Ji‐Ye Huang, Xiao‐Jie Zhao, Xiu‐Lan Sun

**Affiliations:** ^1^ Neuroprotective Drug Discovery Key Laboratory of Nanjing Medical University Nanjing Jiangsu China; ^2^ Jiangsu Key Laboratory of Neurodegeneration Department of Pharmacology Nanjing Medical University Nanjing Jiangsu China

**Keywords:** autophagy, liver kinase B1, microglial polarization, peroxisome proliferator‐activated receptor γ

## Abstract

Microglia‐mediated neuroinflammation plays a dual role in various brain diseases due to distinct microglial phenotypes, including deleterious M1 and neuroprotective M2. There is growing evidence that the peroxisome proliferator‐activated receptor γ (PPARγ) agonist rosiglitazone prevents lipopolysaccharide (LPS)‐induced microglial activation. Here, we observed that antagonizing PPARγ promoted LPS‐stimulated changes in polarization from the M1 to the M2 phenotype in primary microglia. PPARγ antagonist T0070907 increased the expression of M2 markers, including CD206, IL‐4, IGF‐1, TGF‐β1, TGF‐β2, TGF‐β3, G‐CSF, and GM‐CSF, and reduced the expression of M1 markers, such as CD86, Cox‐2, iNOS, IL‐1β, IL‐6, TNF‐α, IFN‐γ, and CCL2, thereby inhibiting NFκB–IKKβ activation. Moreover, antagonizing PPARγ promoted microglial autophagy, as indicated by the downregulation of P62 and the upregulation of Beclin1, Atg5, and LC3‐II/LC3‐I, thereby enhancing the formation of autophagosomes and their degradation by lysosomes in microglia. Furthermore, we found that an increase in LKB1–STRAD–MO25 complex formation enhances autophagy. The LKB1 inhibitor radicicol or knocking down LKB1 prevented autophagy improvement and the M1‐to‐M2 phenotype shift by T0070907. Simultaneously, we found that knocking down PPARγ in BV2 microglial cells also activated LKB1–AMPK signaling and inhibited NFκB–IKKβ activation, which are similar to the effects of antagonizing PPARγ. Taken together, our findings demonstrate that antagonizing PPARγ promotes the M1‐to‐M2 phenotypic shift in LPS‐induced microglia, which might be due to improved autophagy via the activation of the LKB1–AMPK signaling pathway.

## 1 INTRODUCTION

Neuroinflammation has long been known as a pathophysiological process that is related to a number of neurodegenerative disorders, including Alzheimer's disease, Huntington's disease, Parkinson's disease, stroke, depression, and traumatic brain injury (Neupane, 2016; Piirainen et al., 2017; Sampson et al., 2016; Ullah et al., 2017; Xu et al., 2017). Neuroinflammation is triggered by activated and proliferating microglial cells, astrocytes, and other myeloid cells that ultimately produce pro‐inflammatory cytokines, chemokines, and other inflammatory mediators, leading to neuronal damage (Ullah et al., 2017; White, Lawrence, Brough & Rivers‐Auty, 2017). Microglial cells play critical roles in immune surveillance and host defense by acting as the prime resident innate‐immune cells in the central nervous system (CNS; Perry & Holmes, 2014; Salter & Beggs, 2014). Under normal conditions, microglial cells not only provide surveillance of the CNS environment but also respond to danger signals (Crotti & Ransohoff, 2016; Perry & Teeling, 2013). Activated microglial cells undergo morphological transformation (increase in the size of cell bodies and thickness of proximal processes and decreased ramification of distal branches; Plastira et al., 2016; Walker et al., 2014) and secrete pro‐inflammatory cytokines, leading to self‐perpetuating damage to the neurons, also known as the classically activated M1 phenotype. However, an alternatively activated M2 phenotype can be neuroprotective and neurosupportive and can promote recovery. The dichotomy of M1 and M2 is an oversimplified conceptual framework. The status of microglia may include a battery of different but overlapping functional phenotypes. However, the general M1 and M2 classification is nevertheless a useful concept to improve our understanding of microglial functional status during injury progression as well as to help us explore novel therapeutic strategies.

Peroxisome proliferator‐activated receptor γ (PPARγ) is a ligand‐activated transcription factor that belongs to the nuclear receptor family and a master modulator of glucose and lipid metabolism, organelle differentiation, and inflammation (Guo et al., 2017; Zhao et al., 2016). These form heterodimers with the retinoid X receptor and bind to peroxisome proliferator response elements (PPREs) in the promoter region of respective target genes (Hallenborg et al., 2016). Growing evidence indicates that PPARγ agonists efficiently display neuroprotective properties in response to harmful insults, particularly neuroinflammation. Activation of PPARγ by troglitazone and pioglitazone reduces infarct volume by improving neurological function following middle cerebral artery occlusion in rats (Corona & Duchen, 2016; Culman, Zhao, Gohlke & Herdegen, 2007). Also, PPARγ activation by rosiglitazone imparts antidepressant‐ and anxiolytic‐like effects (Guo et al., 2017).

Dysfunction of autophagy and neuroinflammation have been implicated in the pathogenesis of neurodegenerative diseases (Deretic & Klionsky, 2018; Keller & Lunemann, 2018). Autophagic inhibition is involved in lipopolysaccharide (LPS)‐induced microglial activation in cultured primary microglia and the mouse brain (He et al., 2018). Similarly, FAK‐family interacting protein of 200 kDa (FIP200) ablation and autophagy inhibition in neural stem cells lead to their defective differentiation by increased infiltration and activation of microglia (Wang, Yeo, Haas & Guan, 2017). These findings indicate that autophagy deficiency might regulate microglial activation. Although there is growing evidence suggesting that PPARγ is a master regulator of microglial M2 polarization in immune disease, such as multiple sclerosis (MS) and experimental allergic encephalomyelitis (EAE; Kaiser et al., 2009; Orihuela, McPherson & Harry, 2016; Xu & Paul, 2007), the precise mechanisms remain unclear. Therefore, this study aimed to investigate the effects of PPARγ in regulating microglial polarization and to explore the involved mechanisms. Our results revealed that antagonizing PPARγ facilitates the LPS‐induced switch of microglial polarization from the M1 phenotype to the M2 phenotype, which may provide a potent therapeutic strategy for related neuroinflammatory diseases.

## 2 RESULTS

### 2.1 Antagonizing PPARγ using T0070907 promotes M1‐to‐M2 polarization of primary cultured microglia

**Figure 1:**
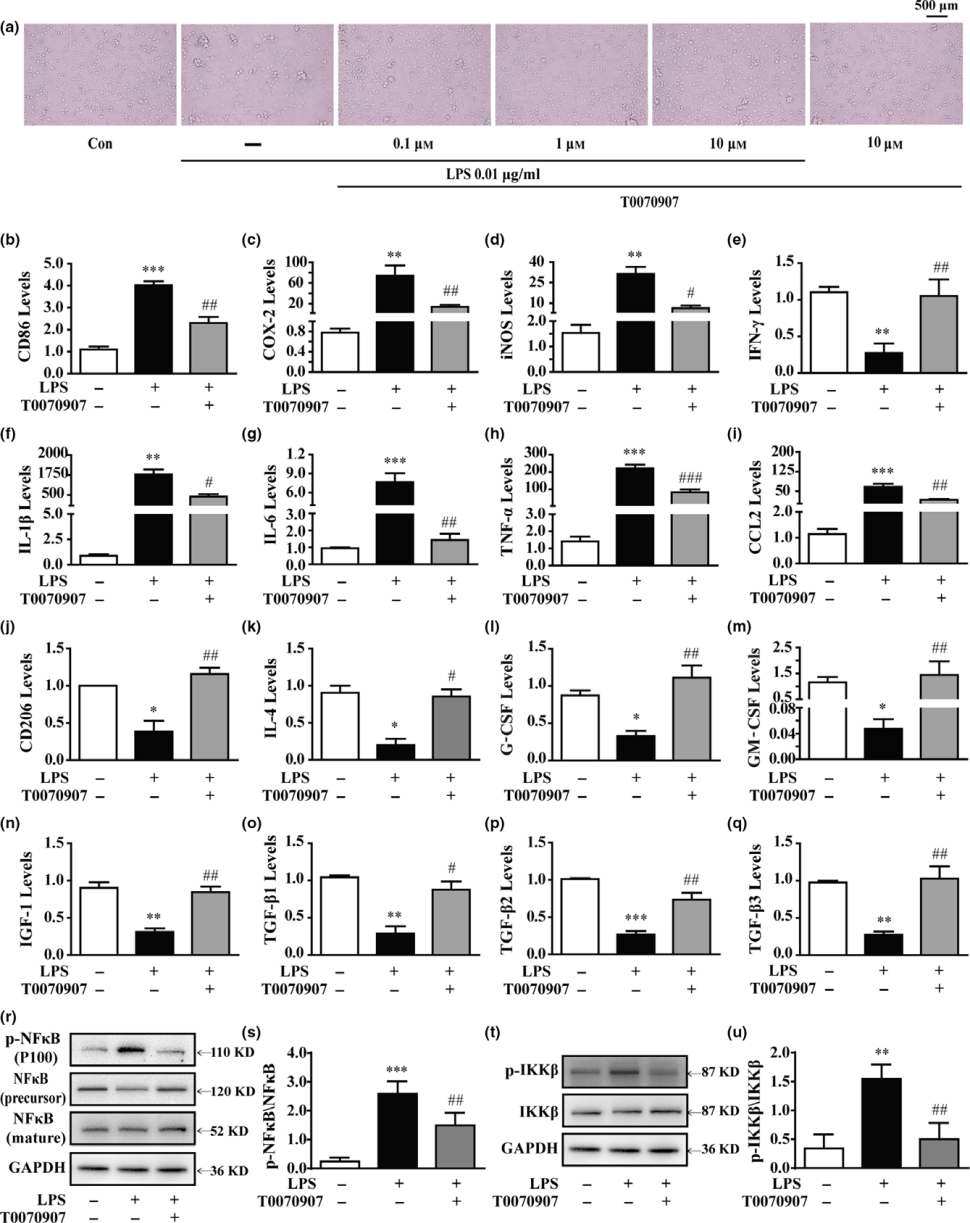
Antagonizing PPARγ prevents LPS‐induced M1 microglial activation and facilitates microglial polarization to the M2 phenotype. Microglia were pretreated 15 min with LPS (0.01 μg/ml) and then incubated with PPARγ antagonist T0070907 (0.1 μm) for 24 hr. (a) The PPARγ antagonist T0070907 suppressed the amoeboid “activated” morphology of microglia induced by LPS. The PPARγ antagonist T0070907 reversed LPS‐induced mRNA expressions of M1 markers (CD86, Cox‐2, iNOS, IFN‐γ, IL‐1β, TNF‐α, and CCL2) (b–i) and mRNA expressions of M2 markers (CD206, IL‐4, G‐CSF, GM‐CSF, IGF‐1, TGF‐β1, TGF‐β2, and TGF‐β3) in microglial cells (j–q). The ratios of p‐NFκB to NFκB (r, s) and p‐IKKβ to IKKβ (t, u) in microglial cells were determined by Western blotting. Data are presented as mean ± SEM,* n* ≥ 4, *p < .05, **p < .01, ***p < .001, compared to the Con group; ^#^ *p* < .05, ^##^ *p* < .01, ^###^ *p* < .001, compared to the LPS group

Figure 1a shows that treatment with PPARγ antagonist T0070907 alone at a concentration of 10 μm did not induce toxicity in microglial cells. However, T0070907 could significantly inhibit LPS‐induced microglial activation. The microglial cells exhibited a decrease in size with retracted branches compared to that in the LPS‐stimulation groups (Figure 1a). Moreover, PPARγ suppression dramatically inhibited the mRNA levels of M1 markers, such as CD86, Cox‐2, iNOS, IL‐1β, IL‐6, TNF‐α, IFN‐γ, and CCL2, compared to those in the LPS groups (Figure 1b–i). Furthermore, the expression of M2 markers, including CD206, IGF‐1, TGF‐β1, TGF‐β2, TGF‐β3, CCL2, CCR2, G‐CSF, and GM‐CSF, increased after T0070907 application (Figure 1j–q). These results demonstrate that antagonizing PPARγ enhances microglial M2 polarization.

NFκB is a key transcription factor that upregulates various pro‐inflammatory mediators, and it is essential for both M1‐ and M2‐like microglial differentiation. After LPS stimulation, a significant increase in the phosphorylation of NFκB and IKKβ was observed (Figure 1r–u). In contrast, antagonizing PPARγ significantly suppressed LPS‐induced phosphorylation of NFκB and IKKβ (Figure 1r–u). These findings suggest that PPARγ suppression participates in the phenotypic shift of microglial polarization by inhibiting the NFκB pathway.

### 2.2 Antagonizing PPARγ reverses LPS‐induced autophagy inhibition in primary cultured microglia

**Figure 2:**
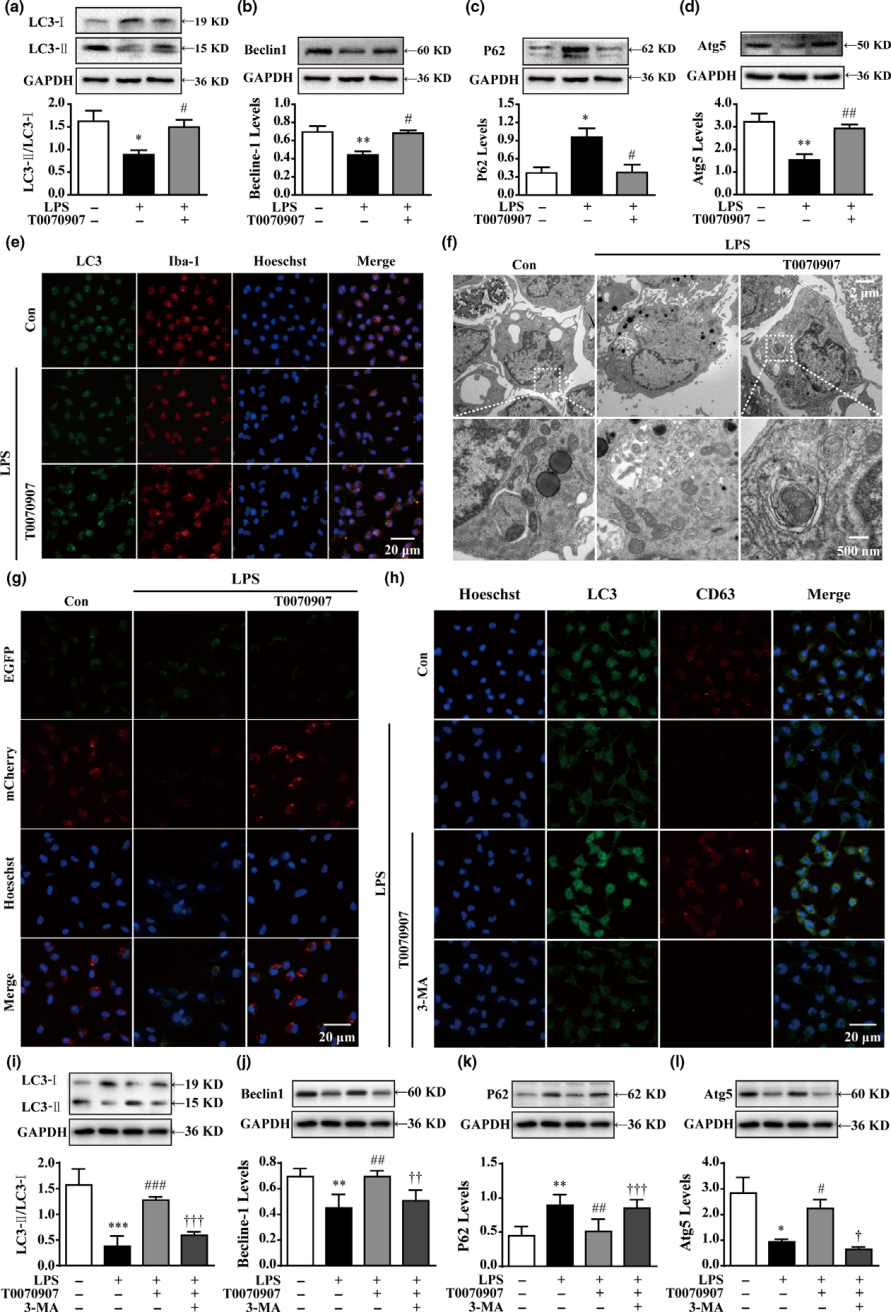
The PPARγ antagonist T0070907 improves autophagy in microglia. (a–d) The PPARγ antagonist T0070907 prevented LPS‐induced decreases in LC3‐II/LC3‐I, Beclin1, and Atg5 and LPS‐induced increases in P62. Data are presented as mean ± SEM, n ≥ 4, *p < .05, **p < .01, compared to the Con group; ^#^ *p* < .05, ^##^ *p* < .01, compared to the LPS group. (e) Representative confocal images of LC3 puncta formation; LC3 (green) was colocalized with Iba1 (red) in cells treated with the PPARγ antagonist T0070907 and LPS. Nuclei counterstained with Hoechst 33342. Scale bar = 20 μm. (f) Representative TEM images. Scale bar = 2 μm. High magnification of the boxed areas is shown below. Scale bar = 500 nm. Autophagosomes (blue arrows), autolysosomes (red arrows), and multilamellar body (yellow arrow). (g) Confocal microscopy analysis was used to measure autophagosomes and autolysosomes by monitoring the distribution and alteration of mCherry and EGFP fluorescent signals from mCherry‐EGFP‐LC3B. Scale bar = 20 μm. Microglia were pretreated with 3‐MA (500 μm) or vehicle for 15 min, followed by treatment with 0.01 μg/ml LPS and 0.1 μm PPARγ antagonist T0070907 for 24 hr. (h) Immunofluorescence staining of LC3 (green), CD63 (red), and nuclei (blue) in LPS, LPS+T0070907, and LPS+T0070907 + 3‐MA groups. Scale bar = 20 μm. The protein expressions of LC3 (i), Beclin1 (j), p62 (k), and Atg (l) were determined by Western blotting. Data are presented as mean ± SEM,* n* ≥ 4, *p < .05, **p < .01, ***p < .001, compared to the Con group; ^#^ *p* < .05, ^##^ *p* < .01, ^###^ *p* < .001, compared to the LPS group; ^†^ *p* < .05, ^††^ *p* < .01, ^†††^ *p* < .001, compared to the LPS+T0070907 group

Numerous studies have shown the important roles of autophagy in microglial inflammation and phenotype shift (Liu et al., 2015; Yang et al., 2014; Zhang, Guo, Zhao, Shao & Zheng, 2016). However, whether PPARγ alters LPS‐induced microglial polarization by modulating autophagy remains unclear. As the conversion of nonlipidated LC3‐I to lipidated LC3‐II is a classical marker of autophagic activity (Mizushima & Yoshimori, 2007), the ratio of LC3‐II/LC3‐I was determined by Western blotting to demonstrate the effects of the PPARγ antagonist on autophagy. LC3‐II expression was enhanced in cells cotreated with T0070907 and LPS, and it was higher than that in the cells treated with LPS alone (Figure 2a). Double immunofluorescence further demonstrated an increase in the accumulation of immunostained LC3‐II puncta (green), which colocalized with Iba1 (red) in the cytoplasm of T0070907 + LPS‐treated cells (Figure 2e). Similar to the LC3‐II level, the levels of two important participants in the formation of autophagosomes, Beclin1/Atg6 and Atg5, significantly increased following cotreatment with PPARγ antagonist T007090 and LPS (Figure 2b,d). LPS treatment significantly increases p62 (also known as SQSTM1, a protein substrate for autophagy used for monitoring autophagic turnover) in microglial cells (Ha et al., 2015), reflecting impaired autophagy. However, T0070907 significantly decreased p62 expression in LPS‐treated microglial cells (Figure 2c). In addition, higher levels of autophagosomes or lysosomes in PPARγ antagonist T0070907‐treated microglial cells were observed by transmission electron microscopy (TEM; Figure 2f).

Complete autophagy requires the fusion of autophagosomes with lysosomes. To further evaluate the impact of T0070907 on autophagic flux, we transfected a tandem construct mCherry‐EGFP‐LC3B plasmid into microglial cells. In general, LC3 appears as a diffuse pattern in the cytoplasm. Upon activation of autophagy, LC3 undergoes aggregation, generating a punctate pattern. The EGFP signal is sensitive to the formation of low‐pH autolysosomes where it is lost, whereas mCherry is more stable. Therefore, the appearance of red dots indicates activated autophagic flux, which is characterized by the successful fusion of autophagosomes with lysosomes. However, the yellow punctum, which is colocalized in both EGFP and mCherry fluorescence, indicates a compartment that has not fused with a lysosome. Figure 2g shows faint mCherry fluorescent signals in LPS‐treated microglial cells, indicating blockage of autophagic flux under LPS‐induced microglial M1 polarization. Similarly, a lower number of colocalization sites for LC3 and the lysosomal marker CD63 were observed in the LPS‐treated group compared to normal conditions (Figure 2h). However, as for the cotreatment with the PPARγ antagonist T0070907, the microglial cells clearly showed the characteristic LC3 punctate staining with red mCherry, but not green EGFP fluorescence (Figure 2g). Furthermore, T0070907 promoted LC3 puncta accumulation and CD63 staining in LPS‐treated microglial cells (Figure 2h). These results suggest that antagonizing PPARγ enhances autophagic flux in microglial cells.

The application of an inhibitor of autophagy, 3‐MA, resulted in a marked reduction in T0070907‐induced LC3 puncta accumulation and colocalization of LC3 puncta and CD63‐positive compartments compared to T0070907 treatment alone (Figure 2h). In addition, Western blot analysis showed that T0070907 induced an increase in the autophagic protein levels, which were significantly reduced by 3‐MA treatment (Figure 2i–l). Taken together, these findings strongly indicate that the enhancement of autophagy by antagonizing PPARγ plays a significant role in microglial M2 polarization.

### 2.3 Liver kinase B1 (LKB1) activation is necessary for PPARγ‐mediated regulation of adenosine 5′‐monophosphate (AMP)‐activated protein kinase (AMPK) phosphorylation in primary cultured microglial cells

**Figure 3:**
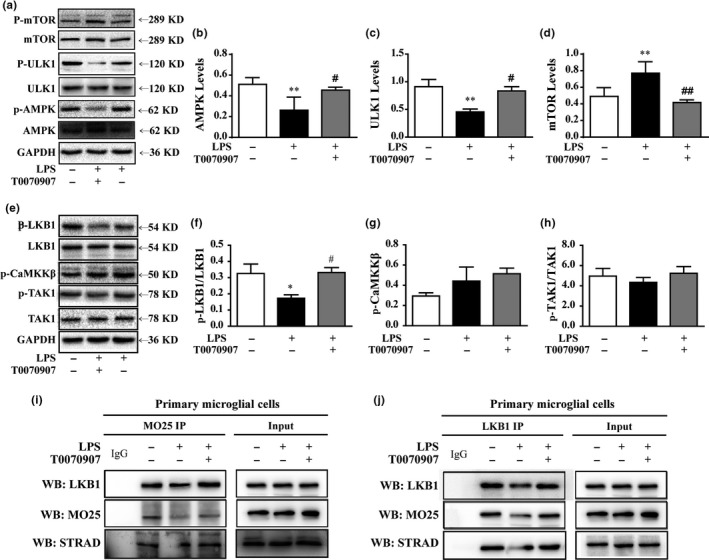
Antagonizing PPARγ reverses LPS‐mediated inhibition of autophagy in microglial cells by activating AMPK. Microglial cells were pretreated with 0.01 μg/ml LPS, followed by treatment with 0.1 μm PPARγ antagonist T0070907 for 24 hr. The upstream regulatory protein levels of autophagy, AMPK (a, b), ULK1 (a, c), and mTOR (a, d) were analyzed by Western blotting. Data are presented as mean ± SEM,* n* ≥ 4, *p < .05, **p < .01, ***p < .001, compared to the Con group; ^#^ *p* < .05, ^##^ *p* < .01, ^###^ *p* < .001, compared to the LPS group; ^†^ *p* < .05, ^††^ *p* < .01, ^†††^ *p* < .001, compared to the LPS+T0070907 group. (e–h) The PPARγ antagonist T0070907 increased the phosphorylation of LKB1, but it did not change the phosphorylation of CaMKKβ and TAK1 in the microglial cells treated with LPS. (i) An anti‐LKB1 antibody was used for Dynabeads Protein G immunoprecipitation, and it detected the immunoprecipitates of MO25 and STRAD by Western blotting. (j) An anti‐MO25 antibody was used for Dynabeads Protein G immunoprecipitation, and it detected the immunoprecipitates of LKB1 and STRAD by Western blotting

As a regulator of autophagy (Kim et al., 2013; Mack, Zheng, Asara & Thomas, 2014; Shang & Wang, 2011), AMPK phosphorylation was found to be reduced in microglial cells after LPS exposure. Furthermore, the increase in AMPKα2 expression and reduction in p‐AMPK protein were restrained by the PPARγ antagonist T0070907 (Figure 3a,b). As phosphorylated mammalian target of rapamycin (mTOR) and unc‐51‐like autophagy activating kinase 1 (ULK1) are involved in the AMPK controlling autophagic process, we examined their phosphorylation in the presence of the PPARγ antagonist. Figure 3a,c,d shows that T0070907 not only increases the phosphorylation of ULK1, but also decreases the phosphorylation of mTOR in LPS‐treated microglia.

LKB1, calmodulin‐dependent protein kinase kinase β (CaMKKβ), and transforming growth factor β‐activated kinase (TAK1) are upstream kinases that can activate AMPK by phosphorylating Thr172, which is situated in the activation loop of the α‐subunit (Jeon, 2016). In our study, we found that LPS stimulation inhibits LKB1 phosphorylation, but it does not affect CaMKKβ and TAK1 phosphorylation. Accordingly, the application of the PPARγ antagonist induced the phosphorylation of LKB1 (Figure 3e–h). LKB1 binds with pseudokinase Ste20‐related adaptor (STRAD) and scaffolding‐like adaptor protein mouse protein 25 (MO25) to form a complex, and it subsequently achieves full activation (Lin et al., 2015; Mihaylova & Shaw, 2011). We further demonstrated the impact of PPARγ antagonist on LKB1 activation using a co‐immunoprecipitation (Co‐IP) assay. The results showed that LPS reduced the binding capacity of LKB1, STRAD, and MO25 in microglial cell lysates without affecting their protein expression levels. Conversely, PPARγ antagonization facilitated the formation of the LKB1–STRAD–MO25 complex (Figure 3i,j). These findings suggest that the phosphorylation of LKB1 is crucial for the regulatory effects of PPARγ on autophagy.

### 2.4 Antagonizing PPARγ enhances autophagy in primary cultured microglia via LKB1 activation

**Figure 4:**
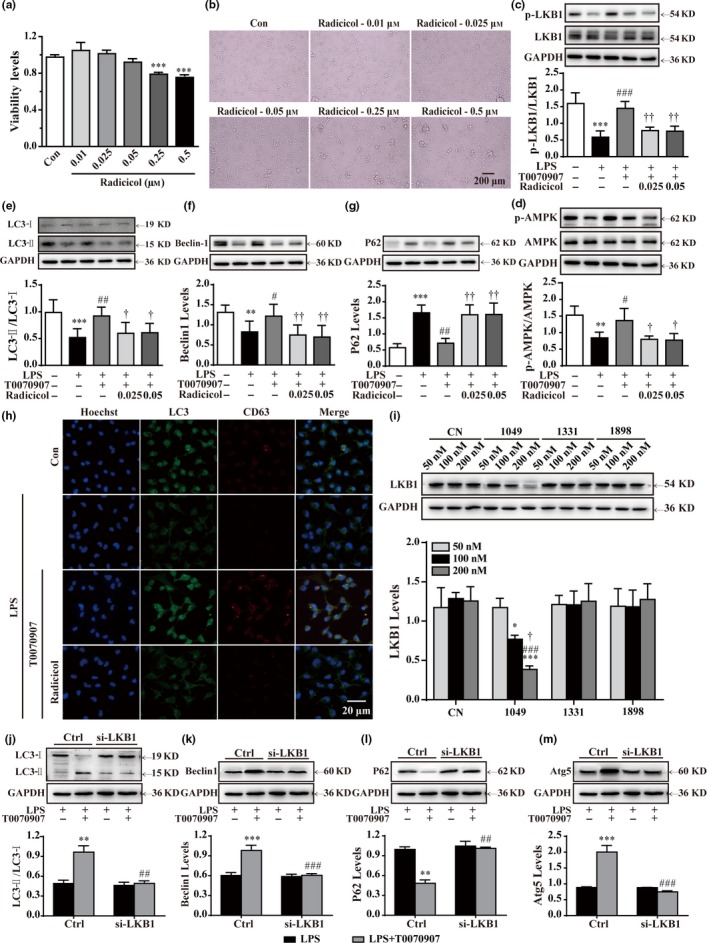
Phosphorylation of LKB1–AMPK is necessary for T0070907‐mediated upregulation of autophagy. (a, b) Below the concentration of 0.05 μm, radicicol did not influence the viability of the microglia**.** After LPS stimulation for 15 min, the LKB1 inhibitor radicicol (0.025 and 0.05 μm) was applied for 15 min before treatment with PPARγ antagonist T0070907 for 24 hr. (c–g) Radicicol turned over the protein expressions of LKB1, AMPK, LC3, Beclin1, and p62 in the microglia treated with LPS and T0070907. Data are presented as mean ± SEM,* n* ≥ 4, *p < .05, **p < .01, ***p < .001, compared to the Con group; ^#^ *p* < .05, ^##^ *p* < .01, ^###^ *p* < .001, compared to the LPS group; ^†^ *p* < .05, ^††^ *p* < .01, compared to the LPS+T0070907 group. (h) Immunofluorescence staining with of LC3 (green), CD63 (red), and nuclei (blue) in the LPS, LPS+T0070907, and LPS+T0070907 + radicicol groups. Scale bar = 20 μm. (i) Quantitation of Western blotting data showing declines in LKB1, which was used to observe the efficiency of transfection. Microglia were pretreated with si‐LKB1 (500 nm) or vehicle for 24 hr and then stimulated with LPS for 15 min and treated with PPARγ antagonist T0070907 for 24 hr. LKB1 siRNA prevented T0070907‐induced increases in LC3‐II/LC3‐I (j), Beclin1 (k), and Atg5 (m) and T0070907‐induced decreases in P62 (l). Data are presented as mean ± SEM,* n* ≥ 4, **p < .01, ***p < .001 compared to the LPS group in the Ctrl; ^##^ *p* < .01, ^###^ *p* < .001, compared to the LPS+T0070907 group in the Ctrl

Radicicol, an inhibitor of LKB1, was used to further elucidate the role of LKB1 in T0070907‐induced AMPK activation and autophagy. Our results showed that radicicol reduced the viability of microglial cells at the concentrations of 0.25 and 0.5 μm (Figure 4a,b). Therefore, radicicol at concentrations of 0.025 and 0.05 μm was used to inhibit LKB1 kinase activity in the following experiments (Figure 4c), and T0070907‐induced AMPK activation was suppressed in microglial cells when treated with radicicol (Figure 4d). Furthermore, we found that treatment with radicicol resulted in a decrease in Beclin1 production and an increase in p62/SQSTM1 expression in microglial cells (Figure 4e,f). In addition, a reduction in immunofluorescence staining of LC3‐II and the conversion of LC3‐I to LC3‐II were observed in radicicol‐treated microglial cells (Figure 4e). Moreover, radicicol treatment blocked T0070907‐induced autophagosome–lysosome fusion, resulting in a significant reduction in colocalization of LC3 puncta and CD63‐positive compartments (Figure 4h). To rule out off‐target effects, primary microglial cells were transfected with LKB1 siRNA to observe the effects of T0070907‐enhanced autophagy. LKB1 expression levels in normal microglial cells were set to 1.0, and relative expression levels of cells after transfection with 50, 100, and 200 μm siRNA‐FM were respectively measured. Compared to siRNA‐LKB1 (1331), siRNA‐LKB1 (1898) and siRNA‐PPARγ (1049) induced a significant decrease in LKB1 expression (60%) at a concentration of 200 nm (Figure 4i). Thus, LKB1 siRNA (1049) was selected for transfection in the subsequent experiments. Our results showed that LKB1 knockdown prevented the T0070907‐induced increase in LC3‐II/LC3‐I, Beclin1/Atg6, and Atg5 and reduction in p62/SQSTM1 (Figure 4j‐m). Collectively, our data indicate that PPARγ antagonist‐mediated autophagy is dependent on the LKB1–AMPK signaling pathway.

### 2.5 Antagonizing PPARγ facilitates primary microglial M1‐to‐M2 polarization via LKB1 activation

**Figure 5:**
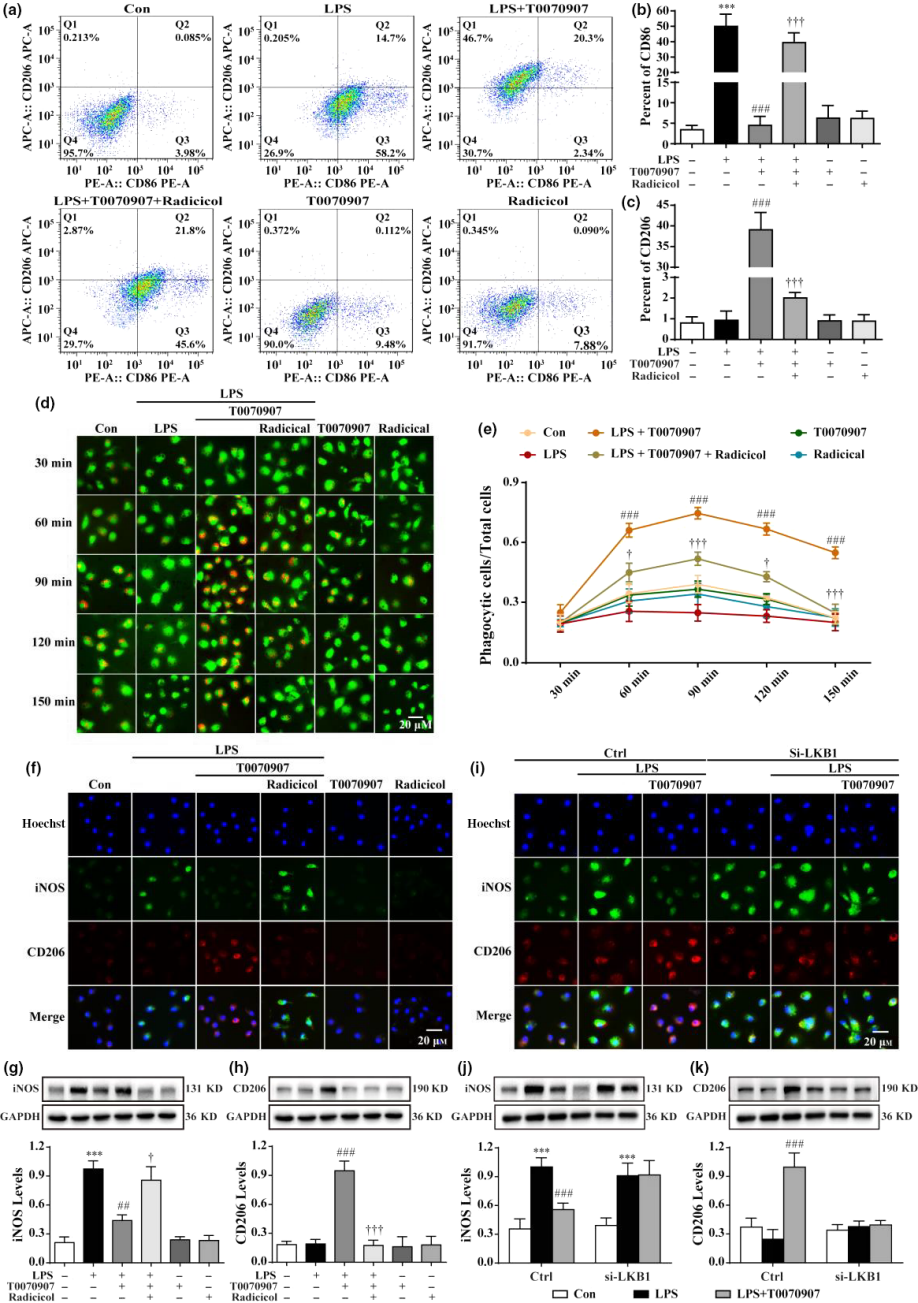
Blocking LKB1 reverses the T0070907‐mediated transition between M1 and M2 phenotypes by suppressing NFκB. After LPS stimulation for 15 min, the LKB1 inhibitor radicicol (0.025 and 0.05 μm) was applied 15 min before treatment with PPARγ antagonist T0070907 for 24 hr. (a) Fluorescence‐activated cell sorting analysis of the microglia in the LPS, LPS+T0070907, and LPS+T0070907 + radicicol groups. Surface expression of CD86 and CD206 was detected in microglia by flow cytometry. The percentage of CD86 (b) and CD206 (c) cells in the microglia was determined. pHrodo^™^ Green Zymosan BioParticles were added to the cells and imaged after 30, 60, 90, 120, and 150 min. The green staining in the microglial cells was due to Cell Tracker^™^ Green. (d) The microglial cells showed the time course of red fluorescence increased, documenting the accumulation of pHrodo‐conjugated zymosan bioparticles (1 μm in diameter) in the intracellular acidic environment corresponding to phagosomes. (e) The proportion between the red‐stained cells and the total cells was calculated. Data are presented as mean ± SEM,* n* = 3, ^###^ *p* < .001 compared to the LPS group in each time point; ^†^ *p* < .05, ^†††^ *p* < .001, compared to the LPS+T0070907 group in each time point. Nonfluorescence appeared at a neutral pH outside of the cell. Scale bar = 20 μm. Radicicol (f) and knocking down LKB1 (i) reversed the PPARγ antagonist T0070907‐induced changes in the protein expression of the M1 markers (iNOS, green) and the M2 markers (CD206, red) by immunofluorescence staining. Scale bar = 20 μm. (g, h) Western blotting showed that radicicol prevented the decreases in iNOS and the increases in CD206 induced by T0070907. Data are presented as mean ± SEM,* n* = 4, ***p < .001, compared to the Con group; ^##^ *p* < .01, ^###^ *p* < .001, compared to the LPS group; ^†^ *p* < .05, ^†††^ *p* < .001, compared to the LPS+T0070907 group. (j and k) Knocking down LKB reversed the PPARγ antagonist T0070907‐induced changes in the protein expressions of iNOS and CD206 by Western blotting. Data are presented as mean ± SEM,* n* = 4, ***p < .001, compared to Con group, respectively in Ctrl or in si‐LKB1; ^###^ *p* < .001, compared to LPS group, respectively in Ctrl or in si‐LKB1

The expression of CD86 and CD206 was used to quantify M1 and M2 microglia by flow cytometry. Figure 5a–c shows that the application of LPS results in a significant decrease in the number of CD206^+^ cells and an increase in the number of CD86^+^ cells in the microglia. The administration of a PPARγ antagonist led to an increase in the number of CD206^+^ cells and a decrease in the number of CD86^+^ cells in LPS‐treated microglia, which was abolished by radicicol treatment. To further confirm the phagocytic function of microglia, the microglial cells were stained green, and pH‐sensitive red dye zymosan bioparticles were used to detect microglial phagocytosis. The red fluorescence did not change in LPS‐treated microglia. However, the red fluorescence continuously increased after treatment with T0070907, reaching an almost 10‐fold increase at 90 min after administration. Radicicol inhibited the T0070907‐induced enhancement of phagocytosis (Figure 5d,e). Accordingly, radicicol reversed the decrease in inducible nitric oxide synthase (iNOS) (green fluorescence, M1 marker) and the increase in CD206 (red fluorescence, M2 marker) induced by the PPARγ antagonist (Figure 5e). Consistently, Western blotting analysis showed that radicicol reversed the T0070907‐induced changes of the expressions of iNOS and CD206 (Figure 5g,h). Furthermore, immunofluorescence revealed that knocking down LKB1 also reversed the T0070907‐induced effects of promoting a M1‐to‐M2 shift (Figure 5f), which was confirmed by Western blotting analysis (Figure 5j,k). Taken together, these results confirm that LKB1 activation is necessary for antagonizing the PPARγ‐mediated M2 polarization of microglia.

### 2.6 PPARγ knockdown activates LKB–AMPK signaling and inhibits LPS‐induced M1 polarization in BV2 cells

**Figure 6:**
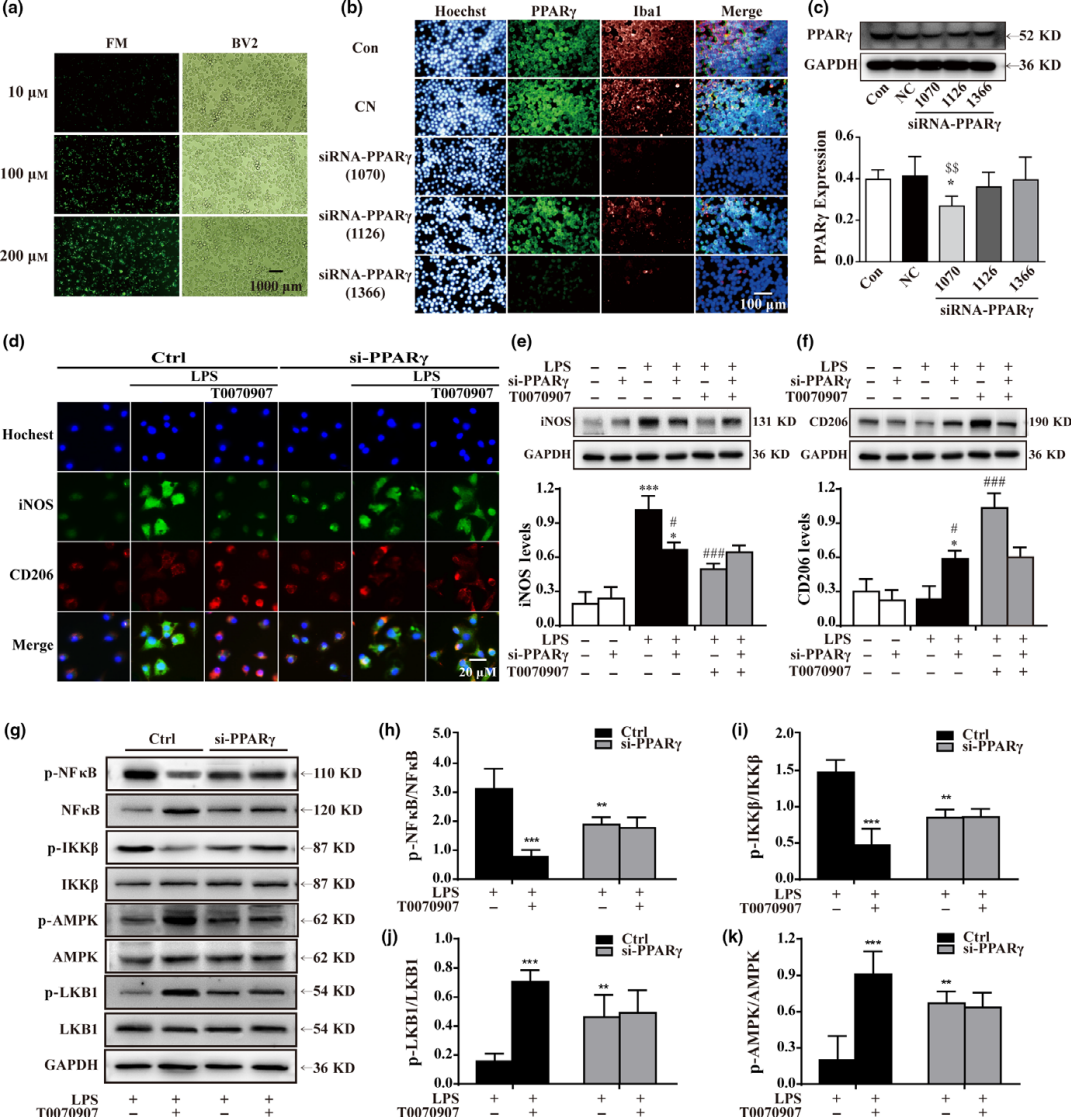
Knocking down PPARγ reduces the inflammatory response by promoting LKB–AMPK activation. (a) Labeled (FM) with green were used to observe the efficiency of transfection reagent in BV2 cells. Scale bar = 1,000 μm. (b) Images of dissociated microglial cells exposed to scrambled (Con, top panels) or PPARγ siRNA, and labeled with antibodies to Iba1 (red) and PPARγ (green), and colabeled with a nuclear stain (blue). Scale bar = 100 μm. (c) Quantitation of Western blotting data showing declines in PPARγ. Statistical analysis was performed using one‐way ANOVA followed by Bonferroni's post hoc test. Data are presented as mean ± SEM,* n* = 5, ^$$^ *p* < .01 compared to the NC group. Microglia were pretreated with si‐PPARγ (500 nm) or vehicle for 24 hr and then stimulated with LPS for 15 min and treated with PPARγ antagonist T0070907 for 24 hr. (d) PPARγ antagonist T0070907 did not influence PPARγ siRNA‐mediated protein expressions of the M1 marker (iNOS, green) and the M2 marker (CD206, red). Scale bar = 20 μm. The experiment was repeated three times. (e, f) Western blotting was used to quantify the expressions of iNOS and CD206 in each group. Data are presented as mean ± SEM,* n* = 4, *p < .05, ***p < .001, compared to the Con group, respectively in Ctrl or in si‐PPARγ; ^###^ *p* < .001, compared to the LPS group, respectively in Ctrl in Ctrl or in si‐PPARγ. PPARγ siRNA inhibited the activation of NFκB and IKKβ (e, f, and g). PPARγ siRNA was also associated with a rise in p‐LKB1 (e, h) and p‐AMPK (e, i). The PPARγ antagonist T0070907 did not affect the efficacy of PPARγ siRNA. Data are presented as mean ± SEM,* n* ≥ 5, **p < .01, ***p < .001, compared to the LPS group in the control

PPARγ expression levels in normal microglial cells were set to 1.0, and the relative expression levels of cells transfected with 10, 100, and 200 μm siRNA‐FM were respectively achieved (Figure 6a), with the most significant reduction (up to 95%) using 200 μm siRNA‐FM. Compared to siRNA‐PPARγ (1126), transfection with siRNA‐PPARγ (1366) and siRNA‐PPARγ (1070) resulted in a 90% suppression of PPARγ expression (Figure 6b,c). siRNA‐PPARγ (1070) induced a lower degree of toxicity in BV2 cells as indicated by Hoechst 33342 staining (Figure 6b). Based on these results, PPARγ siRNA (1070) was used for transfection in the subsequent experiments. Our results showed that knocking down PPARγ inhibited the upregulation of iNOS and downregulation of CD206 in LPS‐treated microglia, which was not affected by treatment with the PPARγ antagonist T0070907 (Figure 6d). Western blotting analysis also showed knocking down PPARγ decreased iNOS expression but increased CD206 expression (Figure 6e,f). In addition, PPARγ knockdown inhibited the activation of the NFκB–IKKβ pathway and enhanced the phosphorylation of LKB1 and AMPK in BV2 cells. The PPARγ antagonist T0070907 did not affect the above regulatory effects of knocking down PPARγ (Figure 6g,k). These results reveal that knocking down PPARγ and antagonizing PPARγ have similar effects in promoting the M1‐to‐M2 phenotypic conversion of microglia.

## 3 DISCUSSION

There is growing evidence suggesting that PPARγ can inhibit microglial activation, promote M2 polarization, and suppress inflammatory cytokines in inflammation‐related diseases, such as MS, EAE, Parkinson's disease (PD), stroke, and major depressive disorder (MDD) (Han et al., 2015; Kaiser et al., 2009; Mandrekar‐Colucci, Karlo & Landreth, 2012; Orihuela et al., 2016; Zhao et al., 2016). Our study also reveals that PPARγ activation could partly suppress LPS‐induced M1 activation, but it does not promote a resting M2 microglial phenotype (Figure S1). However, a number of questions surrounding the role of PPARγ in microglial polarization remain unclear. However, the present study revealed that antagonizing PPARγ also inhibits LPS‐induced microglial activation, and does not alter the expressions of PPARα, PPARβ/δ and PPARγ in microglia (Figure S2). Moreover, the application of the PPARγ antagonist T0070907 facilitates microglial polarization from the M1 to the M2 phenotype.

The specific classification of M1 and M2 polarized microglia remains a topic for debate. M1/M2 polarization of microglia is largely classified by the expression of M1 (e.g., CD86, IL‐1β, TNF‐α, IFN‐γ, Cox‐2, and iNOS)‐ and M2 (e.g., CD206, IGF‐1, TGF‐β, CCR2, G‐CSF, and GM‐CSF)‐associated genes. In response to distinct microenvironmental cues, the polarized M1 or M2 subpopulation can reverse its phenotype and function. Therefore, strategies based on promoting M1‐to‐M2 phenotypic conversion of microglial cells may be potentially utilized in the treatment of neuroinflammation‐induced injury. In the present study, we found that the PPARγ antagonist T0070907 increases the levels of M2 markers, such as CD206, IL‐4, IGF‐1, TGF‐β1, TGF‐β2, TGF‐β3, G‐CSF, and GM‐CSF, and reduces the levels of M1 markers (CD86, Cox‐2, iNOS, IL‐1β, IL‐6, TNF‐α, IFN‐γ, and CCL2). Flow cytometry and immunofluorescence analyses confirmed the effects of T0070907. Moreover, antagonizing PPARγ could enhance microglial phagocytosis. p‐NFkB and p‐IKKβ were downregulated in microglial cells after the application of the PPARγ antagonist T0070907. These data reveal that antagonizing PPARγ promotes M1‐to‐M2 polarization and plays anti‐inflammatory roles in LPS‐stimulated primary microglia.

There is increasing evidence that autophagy is important for the induction and function of M2 phenotype microglia. In the current study, the ratio of LC3‐II/LC3‐I and the expression levels of autophagic markers, including Beclin1 and Atg5, were enhanced by T0070907. Furthermore, we found that T0070907 treatment improved autophagosome formation and the autophagosome–lysosome fusion. Moreover, T0070907 induced the significant colocalization of LC3 puncta and CD63‐positive compartments in microglial cells. 3‐MA blocked the T0070907‐induced enhanced effects of autophagy. Therefore, our results reveal that PPARγ antagonism can enhance autophagy, which contributes to microglial M2 polarization.

AMPK activates autophagy (Hindupur, González & Hall, 2015) by directly or indirectly activating ULK1 (Egan et al., 2011; Kim, Kundu, Viollet & Guan, 2011). AMPK not only directly phosphorylates and activates ULK1 to induce autophagy, but also indirectly activates ULK1 by inhibiting mTORC1, which phosphorylates and inhibits ULK1 to disrupt the AMPK–ULK1 interaction (Inoki, Kim & Guan, 2012). This coordinated regulation of AMPK, mTORC1, and ULK1 eliminates damaged organelles and maintains mitochondrial integrity by initiating autophagy. Herein, our results show that autophagy is enhanced by the PPARγ antagonist by suppressing the levels of p‐mTOR and increasing the expression of p‐AMPK and p‐ULK1. PPARγ knockdown also increases the phosphorylation of AMPK, which coincides with the effects of the PPARγ antagonist T0070907.

AMPK activity is increased when its Thr172 residue in the activation loop is phosphorylated by upstream kinases (Hindupur et al., 2015). In mammals, there are three activating kinases for AMPK, namely LKB1, CaMKKβ, and TAK1. We found that the PPARγ antagonist only promotes LKB1 phosphorylation against LPS‐mediated AMPK inhibition, and no effects on the phosphorylation of CaMKKβ and TAK1 were observed. Additionally, the transfection of cells with PPARγ siRNA also significantly improved p‐LKB1 protein levels. Furthermore, we found that the PPARγ antagonist accelerates the formation of the LKB1–STRAD–MO25 complex. To confirm the crucial roles of LKB1 in PPARγ antagonist‐mediated microglial polarization, radicicol was used to inhibit LKB1 activation. We found that radicicol could potentially inhibit AMPK activity and autophagy. Subsequently, radicicol reversed the M1‐to‐M2 shift and inhibited T0070907‐induced phagocytosis. To rule out the effects of other targets besides LKB1, we used LKB1 siRNA to knock down LKB1 expression in primary microglia. LKB1 knockdown prevented T0070907‐induced increases in LC3‐II/LC3‐I, Beclin1/Atg6, and Atg5 expression, as well as the reduction in p62/SQSTM1 expression. Similar to radicicol, LKB1 knockdown could significantly inhibit T0070907‐mediated increases in the M2 microglial marker (CD206) and reductions in the M1 microglial marker (CD86). These results demonstrate that LKB1 activation is necessary for antagonizing PPARγ‐induced autophagy and the M1‐to‐M2 microglial phenotype shift.

To observe whether the PPARγ antagonist T0070907 has off‐target effects, we used siRNA to knock down PPARγ and then tested the effects of T0070907. We found that knocking down PPARγ could reverse LPS‐mediated activation of NFkB, inhibit LPS‐induced microglial M1 polarization, and promote M2 polarization. The PPARγ antagonist T0070907 did not affect the above regulative effects of PPARγ knockdown, suggesting that T0070907 acts on PPARγ to regulate microglial polarization.

## 4 CONCLUSIONS

Our results reveal that antagonizing PPARγ promotes an M1‐to‐M2 phenotypic shift in LPS‐induced microglia, which might be due to improved autophagy via the activation of the LKB1–AMPK signaling pathway. The present study provides the first evidence for the critical role of the PPARγ antagonist in microglial polarization, as well as providing a new perspective on microglia‐mediated neuroinflammation.

## 5 EXPERIMENTAL PROCEDURES

### 5.1 Primary microglial cell culture

Primary microglial cell cultures were performed as previously described and were isolated from 1‐ to 3‐day‐old postnatal Sprague‐Dawley rats, which were purchased from Shanghai SIPPR‐Bk Laboratory Animal Co. Ltd (Shanghai, China). All of the animal operational procedures were performed in accordance with the Institution for Animal Care and Use Committee and approved by Animal Core Facility of Nanjing Medical University. Briefly, primary cultures of glial cells were obtained from the cerebral cortices, which were earlier digested by 0.25% trypsin/EDTA (Gibco, Grand Island, NY, USA) at 37°C for 20 min and seeded into poly‐d‐lysine‐coated (0.1 mg/ml; Sigma Chemical, St. Louis, MO, USA) 25‐cm^2^ culture flasks. The cultures were maintained for 10 days in Dulbecco's modified Eagle's medium (DMEM) (Gibco) supplemented with 10% heat‐inactivated fetal bovine serum (FBS) (Gibco) at 37°C in a humidified 5% CO_2_–95% air atmosphere. Media were replaced one day after preparation and subsequently every 2–3 days. Microglial cells were separated from the mixed primary culture by flapped for 15 min and then plated in culture vessels with DMEM medium containing 10% FBS, 100 U/ml penicillin, and 100 μg/ml streptomycin. Before the experiments, the percentage of the primary microglial cells was confirmed by Iba1 staining with over 97% purity.

### 5.2 Drugs and treatment

All of the experiments were conducted 24 hr after the cells were seeded. Primary microglial cells were respectively treated with the PPARγ antagonist rosiglitazone (Selleck Chemicals, Houston, TX, USA) or the PPARγ antagonist T0070907 (Selleck Chemicals) diluted in dimethylsulfoxide (DMSO; Sigma) with a final concentration of 0.1 μm, which was applied after the treatment with LPS (0.01 μg/ml; Sigma‐Aldrich, St. Louis, MO, USA). The BV2 cells were passaged before reaching confluence using 0.025% (v/v) trypsin/EDTA in PBS, and they were seeded on poly‐D‐lysine‐coated dishes at a suitable density. Then, 10 μg/ml LPS was applied to differentiated BV2 cells 15 min before the treatment with 10 μm PPARγ antagonist T0070907, and they were then cultured in a humidified 5% CO_2_–95% air environment at 37°C for 24 hr. Radicicol (0.025 μm and 0.05 μm; Selleck Chemicals) and 3‐MA (500 μm; Sigma‐Aldrich) were used as the LKB1 inhibitor and autophagy inhibitor, respectively.

### 5.3 Transfection of primary microglial cells with plasmid

Microglial cells were cultured at the density of 15 × 10^4^ cells per well in a 24‐well plate at 37°C in a 5% CO_2_ humidified atmosphere and grown to 60% confluency. Microglial cells were transfected with plasmid mCherry‐EGFP‐LC3 (Biogot Technology, Co., Ltd.) using Lipofectamine^®^ MessengerMAX^™^ reagent (Invitrogen, NY, USA) according to the manufacturer's instructions.

### 5.4 Western blotting

Cells were lysed in RIPA buffer containing 2 mm phenylmethylsulfonyl fluoride (PMSF). The lysates were centrifuged at 13225 g for 15 min. The protein concentrations were determined with an enhanced BCA Protein Assay kit (Beyotime Biotechnology, Shanghai, China), and the proteins were denaturalized with 5× loading buffer. Total proteins (30 μg) were separated by sodium dodecyl sulfate–polyacrylamide gel electrophoresis (SDS‐PAGE) and transferred to polyvinylidene fluoride (PVDF) membranes (Roche, Mannheim, Germany). Then, 5% skim milk in Tris‐buffered saline–Tween‐20 (TBST) (1 mol/L Tris‐HCl (pH 7.5), 0.8% NaCl, and 0.1% Tween‐20) was used to block the membranes for 1 hr at room temperature. The membranes were then incubated with the following primary antibodies at 4°C overnight: anti‐AMPKα2 (Proteintech, Cat# 18167‐1‐AP, RRID: AB_10695046, 1:1,000); anti‐phospho‐AMPKα (Thr172) (CST, Cat# 2531, RRID: AB_330330, 1:1,000); anti‐LKB1 (CST, Cat# 3047, RRID: AB_2198327, 1:1,000); anti‐phospho‐LKB1 (CST, Cat# 3482, RRID: AB_2198321, 1:1,000); anti‐phospho‐CaMKII (CST, Cat# 12716, RRID: AB_2713889, 1:1,000); anti‐TAK1 (CST, Cat# 5206S, RRID: AB_10694079, 1:1,000); anti‐phospho‐TAK1 (CST, Cat# 9339, RRID: AB_2140096, 1:1,000); anti‐IKKβ (CST, Cat# 8943, RRID: AB_11024092, 1:1,000); anti‐phospho‐IKKβ (CST, Cat# 2697, RRID: AB_2079382, 1:1,000); anti‐NFκB2 P100/P52 (Bioword, Cat# BS1255, RRID: AB_1663263, 1:500), anti‐phospho‐NFκB2 P100 (Ser866/870) (CST, Cat# 4882, RRID: AB_2153403, 1:1,000); anti‐LC3‐I/II (Proteintech, Cat# 14600‐1‐AP, RRID: AB_2137737, 1:1,000); anti‐mTOR (Proteintech, Cat# 20657‐1‐AP, RRID: AB_10734440, 1:1,000); anti‐phospho‐mTOR (Affinity Biosciences, Cat# AF3309, 1:1000); anti‐ULK1 (Abcam, Cat# ab128859, RRID: AB_11156928, 1:1,000); anti‐Atg5 (Proteintech, Cat# 10181‐2‐AP, RRID: AB_2062045, 1:1,000); and anti‐GAPDH (Proteintech, Cat# 10494‐1‐AP, RRID: AB_2263076, 1:5,000). The primary antibody was recycled and membranes were washed four times in Tris‐buffered saline with Tween‐20 (TBST) for 10 min each time. Goat anti‐rabbit IgG (H + L) secondary antibody and HRP secondary antibodies (Thermo Fisher Scientific, Cat# AF3309, AB_228341, 1:10,000) were incubated for 1 hr at room temperature. After the removal of the secondary antibody, the membranes were washed thrice with TBST for 15 min each time. The relative proteins levels were detected by enhanced chemiluminescence reagent and ImageJ software.

### 5.5 RNA extraction

Total RNA was extracted from the cells with different treatments using TRIzol reagent (Invitrogen Life Technologies, CA, USA), according to the protocol provided by the manufacturer. The concentration and purity of the total RNA were measured using a NanoDrop 2000 spectrophotometer (NanoDrop Technologies, Thermo Scientific, USA). A reverse transcription kit was used to synthesize the complementary DNA, and the additional total RNA samples were stored at −80°C.

### 5.6 Reverse transcription and real‐time quantitative PCR

Total RNA (1 μg) was reverse‐transcribed to synthesize the complementary DNA using a PrimeScript^™^ RT Master Mix (TaKaRa, Japan). According to the manufacturer's instructions, the reverse transcription reaction in the thermal cycler (Eppendorf, Germany) was incubated at 37°C for 15 min, activated at 85°C for 5 s, and then held at 4°C.

Quantitative real‐time PCR was performed using SYBR^®^ Premix Ex Taq I (TaKaRa, Japan) to detect the mRNA levels on a QuantStudio 5 Real‐Time PCR System (Applied Biosystems, USA). Each 10 μL reaction contained 1× SYBR Green Master Mix, forward and reverse primers (sequences are listed in Table 1) at 10 μm concentration, and a cDNA sample as the template. The PCR conditions were as follows: 95°C for 30 s, followed by 40 cycles at 95°C for 5 s and 58°C to 60°C for 34 s. The amplification specificity was validated by the presence of a single peak in the melting curves. GAPDH was used as the endogenous control, and the relative expression of the target genes was determined using the 2^(−ΔΔct)^ method. To confirm the results, each sample was run in triplicate, and the experiments were repeated at least thrice.

**Table 1:** Primer sequences using qPCR

Target gene	Forward primer sequence (5′–3′)	Reverse primer sequence (5′–3′)
*CD86*	TAGGGATAACCAGGCTCTAC	CGTGGGTGTCTTTTGCTGTA
Cox‐2	GCAAATCCTTGCTGTTCCAACC	GGAGAAGGCTTCCCAGCTTTTG
*iNOS*	GCAGAATGTGACCATCATGG	ACAACCTTGGTGTTGAAGGC
*IL‐1*β	TGATGTTCCCATTAGACAGC	GAGGTGCTGATGTACCAGTT
*TNF‐*α	GTAGCCCACGTCGTAGCAAA	CCCTTCTCCAGCTGGGAGAC
*IFN‐*γ	GAGGTGAACAACCCACAGA	TATTGGCACACTCTCTACCC
*IL‐6*	TCTTGGGAC‐TGATGCTGGTG	CAGAATTGCCATTGCACAACTC
*CD206*	AGTTGGGTTCTCCTGTAGCCCAA	ACTACTACCTGAGCCCACACCTGCT
*Arg1*	TCACCTGAGCTTTGATGTCG	TTCCCAAGAGTTGGGTTCAC
*IL‐4*	GGTCTCAGCCCCCACCTTGC	CCGTGGTGTTCCTTGTTGCCGT
*IL‐10*	AATTCCCTGGGTGAGAAGCTG	TCATGGCCTTGTAGACACCTTG
*Ym1*	ACCCCTGCCTGTGTACTCACCT	CACTGAACGGGGCAGGTCCAAA
*TGF‐*β*1*	TGAGTGGCTGTCTTTTGACG	GGTTCATGTCATGGATGGTG
*TGF‐*β*2*	CTCCACATATGCCAGTGGTG	CTAAAGCAATAGGCGGCATC
*TGF‐*β*3*	CCCAACCCCAGCTCCAAGCG	AGCCACTCGCGCACAGTGTC
*IGF‐1*	CAGTTCGTGTGTGGACCAAG	GTCTTGGGCATGTCAGTGTG
*CCL2*	TTCACTGGCAAGATGATCCC	TGCTTGAGGTGGTTGTGGAA
*CCR2*	ATGCTGTCCACATCTCGTTCTCG	TTATAAACCAGCCGAGACTTCCTGC
*G‐CSF*	CCATTGTCCATCTTGGGGATC	CCTGGAAGCTGTTGTTCCATG
*GM‐CSF*	TACCACACCCAGCATTCCTCC	GACCCCTCGCCCAGGTACAGT
*GAPDH*	GTTTGTGATGGGTGTGAACC	TCTTCTGAGTGGCAGTGATG

### 5.7 Immunofluorescence staining and immunofluorescence microscopy

Microglial cells were seeded at the density of 15 × 10^4^ cells/well in a 24‐well plate and treated with drugs in complete culture medium. After 24 hr, the culture medium was removed, and after washing twice with PBS, the cells were fixed with 4% paraformaldehyde at room temperature for 30 min and rinsed thrice in PBS for 5 min each time. Then, the cells were blocked in 0.1% Triton X‐100 and 3% BSA in PBS for 1 hr at room temperature and probed with primary antibodies overnight at 4°C. After they were washed, the cells were treated with FITC‐labeled Alexa Fluor‐488‐ and/or Alexa Fluor‐555‐conjugated secondary antibody (Invitrogen, NY, USA) at a 1:2,000 dilution for 1 hr. The cells were counterstained with Hoechst 33342 for 10 min and washed thrice with PBS for 5 min each time. The stained cells were visualized and photographed using a confocal microscope (Nikon A1RSi, Tokyo, Japan).

### 5.8 Laser scanning confocal microscopy

Confocal microscopy was used to measure autophagosomes and autolysosomes by monitoring the distribution and alteration of mCherry and EGFP fluorescent signals from mCherry‐EGFP‐LC3B in microglial cells treated with LPS and/or T0070907.

### 5.9 Co‐IP assay

Microglial cells were grown to 80%–90% confluency in a six‐well plate and treated with LPS (0.01 μg/ml) and the PPARγ antagonist T0070907 (0.1 μm). After 24 hr, the cells were washed twice with cold PBS, and 400 μL NP‐40 RIPA lysis buffer (Shanghai, China) containing a proteinase inhibitor cocktail (Roche, Mannheim, Germany) was added to four wells of the six‐well plate. After centrifugation at 13225 g for 15 min at 4°C, the protein concentrations of the cell lysates were determined using an enhanced BCA protein assay kit (Beyotime Biotechnology, Shanghai, China). For immunoprecipitation, 500 μg of protein was incubated overnight at 4°C on a rotating device with the following primary antibodies: anti‐LKB1 (Proteintech, Chicago, IL, USA; 1:200), anti‐MO25 (CST, MA, USA; 1:50 dilution), and rabbit IgG (Proteintech; 1:200 dilution). Then, 20 μL of a protein A/G Plus‐Agarose immunoprecipitation reagent (Santa Cruz) was used to capture the conjugated polymers at 4°C on a rotating device for 1 hr. Immunoprecipitates were collected by centrifugation at 547 g for 5 min at 4°C and washed with 1 ml of RIPA buffer containing PMSF, and this was repeated four times. The supernatant was aspirated and discarded, and the resuspended pellet was denatured with 5× loading buffer. After boiling, the immunoprecipitated complexes were separated by SDS‐PAGE and analyzed by Western blotting using the following specific antibodies: anti‐LKB1 (Proteintech; 1:1,000 dilution), anti‐MO25 (CST, MA, USA; 1:1,000 dilution), and anti‐STRAD (Abcam, Chicago, IL, USA; 1:2,000 dilution) as described previously.

### 5.10 TEM analysis

To observe the autophagy, the ultrastructural analysis was performed as described previously (Liu et al., 2016). Briefly, microglial cells were fixed with ice‐cold glutaraldehyde (2.5% in 0.1 m cacodylate buffer, pH 7.4) for 30 min, postfixed in OsO_4_, and embedded in epoxy resin. Ultrathin sections (70–80 nm) were stained with uranyl acetate/lead citrate and examined in an electron microscope (Hitachi 600; Hitachi, Tokyo, Japan). For quantification of autophagic vacuoles, the number of autophagosomes per optical field using 30 optical fields from each sample was calculated.

### 5.11 Phagocytosis

Primary microglial cells were plated on a poly‐(l‐lysine)‐coated confocal plate, incubated overnight in culture medium, and then processed according to the pHrodo^™^ Red zymosan bioparticle procedure of Invitrogen (Carlsbad, CA, USA). After two washes with PBS, the PBS was added with 1 μm Cell Tracker^™^ Green for 20 min. After two washes with PBS, these were incubated at 37°C in the Opti‐MEM medium containing 10 μl/ml of pHrodo Red zymosan bioparticles with or without LPS (0.01 μg/ml) and/or T0070907 (0.1 μm) and/or radicicol (0.05 μm) for 15 min. The treated microglia were examined every 30 min by confocal microscopy (Nikon A1RSi, Tokyo, Japan).

### 5.12 Transfection of primary microglial cells with siRNA

Primary microglia were seeded at the density of 120 × 10^4^ cells per well in six‐well plates and transfected with siRNAs (50 nm, 200 nm, and 500 nm) targeting LKB1 using Lipofectamine^®^ MessengerMAX mRNA Transfection Reagent (Invitrogen, NY, USA) according to the manufacturer's instructions (sequences are listed in Table 2). More than 90% knockdown of the targeted proteins was observed after 500 nm siRNA treatment. Scrambled siRNA and target gene‐specific siRNAs were purchased from GenePharma (Shanghai, China).

**Table 2:** Primer sequences of siRNAs

Target gene	Sense (5’–3’)	Antisense (5’–3’)
Negative control FAM	UUCUCCGAACGUGUCACGUTT	ACGUGACACGUUCGGAGAATT
LKB1‐1049	CGGUCAAGAUCCUCAAGAATT	UUCUUGAGGAUCUUGACCGTT
LKB1‐1331	AGGGCAUUGUUCACAAGGATT	UCCUUGUGAACAAUGACCUTT
LKB1‐1898	GAGGACGGCAUCAUCUAUATT	UAUAGAUGAUGCCGUCCUCTT

### 5.13 Flow cytometry

The microglia were collected and washed twice with PBS, and blocked by 0.1% Triton X‐100 and 3% BSA in PBS. The microglia were then stained with CD86‐FITC (rat) and CD206‐PE (rat) antibodies (BD Biosciences, La Jolla, CA, USA), respectively. The microglia were examined with a BD FACSVerse flow cytometer (BD Biosciences), and all of the tests were controlled by the homologous isotype control antibodies.

### 5.14 Transfection of BV2 cells with siRNA

BV2 microglial cells were provided by Shanghai Institute of Materia Medica (Shanghai, China) and cultured in DMEM supplemented with 10% FBS, 100 U/ml penicillin, and 100 μg/ml streptomycin in a humidified 5% CO_2_–95% air environment at 37°C.

BV2 cells were seeded at the density of 30 × 10^4^ cells per well in six‐well plates and transfected with siRNAs (10, 100, and 200 nm) targeting PPARγ using Lipofectamine^®^ 2000 reagent (Invitrogen, NY, USA) according to the manufacturer's instructions (sequences are listed in Table 3). More than 90% knockdown of the targeted proteins was observed after 500 nm siRNA treatment. Scrambled siRNA and target gene‐specific siRNAs were purchased from GenePharma (Shanghai, China).

**Table 3:** Primer sequences of siRNAs

Target gene	Sense (5′–3′)	Antisense (5′–3′)
Negative control FAM	UUCUCCGAACGUGUCACGUTT	ACGUGACACGUUCGGAGAATT
PARγ‐1070	GCGGAGAUCUCCAGUGAUATT	UAUCACUGGAGAUCUCCGCTT
PPARγ‐1126	CCUGGCAAAGCAUUUGUAUTT	AUACAAAUGCUUUGCCAGGTT
PPARγ‐1366	GCAAGAGAUCACAGAGUAUTT	AUACUCUGUGAUCUCUUGCTT
GAPDH‐420	CACUCAAGAUUGUCAGCAATT	UUGCUGACAAUCUUGAGUGAG

### 5.15 Statistical analysis

The obtained data are presented as mean ± SEM of at least three independent experiments. The relationship between two factors was analyzed using Pearson correlation analysis, and bootstrapping was used in paired‐samples tests. Groups were compared using a two‐way ANOVA with post hoc Bonferroni's multiple comparisons test and one‐way ANOVA with post hoc Tukey's multiple comparisons test. All of the data were analyzed with GraphPad Prism 6.0 software. A value of *p* < .05 indicated that the difference was statistically significant.

## DISCLOSURE

The authors declare that they have no conflict of interests to report.
