# Correction to “Antagonizing Peroxisome Proliferator‐Activated Receptor γ Facilitates M1‐To‐M2 Shift of Microglia by Enhancing Autophagy via the LKB1‐AMPK Signaling Pathway”

**DOI:** 10.1111/acel.70025

**Published:** 2025-02-27

Ji, Juan, Xue, Teng Fei, Guo, Xu Dong, Yang, Jin, Guo, Ruo Bing, Wang, Juan, Huang, Ji Ye, Zhao, Xiao Jie, Sun, Xiu Lan. Antagonizing Peroxisome Proliferator‐Activated Receptor Gamma Facilitates M1‐To‐M2 Shift of Microglia by Enhancing Autophagy via the LKB1‐AMPK Signaling Pathway. Aging Cell 2018;17(4): 1–16.

In the published version of the above article, we noticed the following errors in Figure 4b and in Figure 2e.

1. Collection and production of the images in Figure 4b were done by two different people. Due to unclear communication between the two, pictures other than those of this experiment were imported when preparing Figure 4b. Recently, while reviewing our previous data to start new work, our team spotted the error in Figure 4b. Figure 4b is designed to supplement Figure 4a, aiming to demonstrate that radicicol decreases the viability of microglia at concentrations of 0.25 and 0.5 μm. We solemnly declare that the alteration in Figure 4b has no impact on our research results and conclusions. The corrected figure is provided below.

**Figure:**
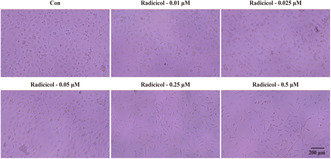
Figure 4b

2. In Figure 2e, we found that the Hoeschst blue fluorescence image of the LPS group was wrongly placed.

We regret that an inadvertent mistake occurred in the process of assembling images. This correction does not impact the findings and conclusions of the paper. We would like to assure the readers that the corrected image does not alter the validity of the research. The corrected figure is provided below.

**Figure:**
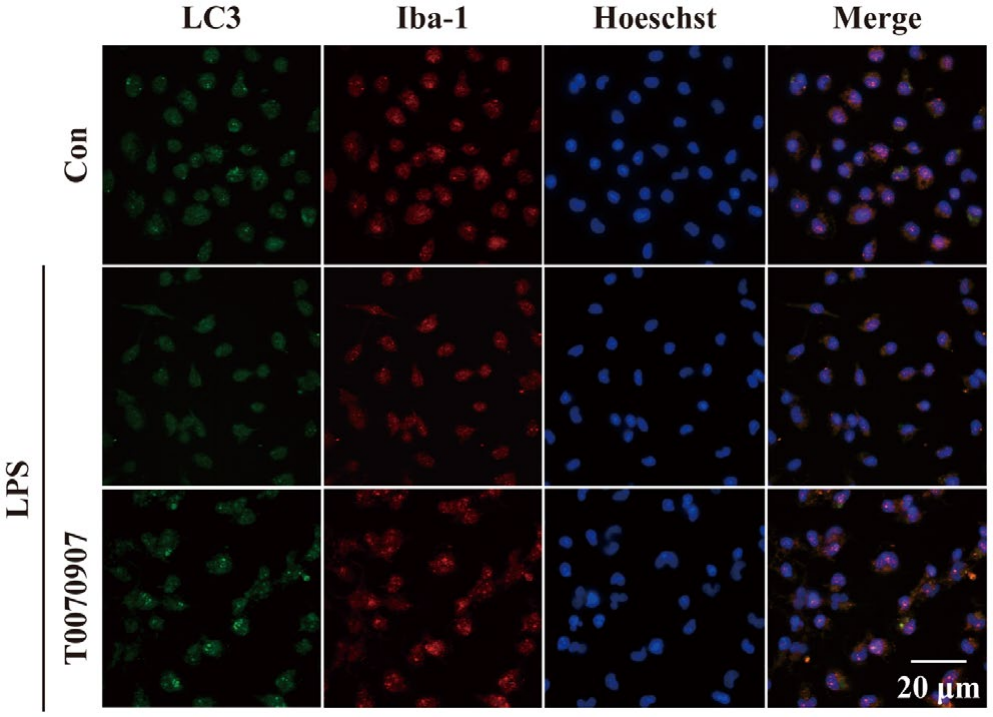
Figure 2e

