# Supplementary material for: Antagonizing peroxisome proliferator‐activated receptor γ facilitates M1‐to‐M2 shift of microglia by enhancing autophagy via the LKB1–AMPK signaling pathway
Source: Aging Cell. 2018 May 8;17(4):e12774. doi: 10.1111/acel.12774 (PMC6052482; doi:10.1111/acel.12774)
Supplement: Supplementary file 1 [file ACEL-17-na-s001.docx]

**Supplementary Materials**

**Activating PPARγ by rosiglitazone inhibits LPS-induced microglial activation**

LPS (0.01 μg/ml) induced microglial cells activation which showed larger and rounder shape with slim branches compared with the control group. LPS at the concentrations of 1 and 10 μg/ml showed cytotoxic effect, and decreased the microglial population. To determine the impact of PPARγ activation on LPS-mediated microglial activity and M1/M2 polarization, we used PPARγ agonist rosiglitazone (0.1, 1, 10 μM) to treat with miacroglial cells, and found PPARγ receptor activation did not affect LPS-mediated microglial activation and morphological change. LPS-stimulation dramatically increased gene expression of known cytotoxic M1 markers (CD86, COX-2, iNOS, IL-1β, IL-6, TNF-α, IFN-γ and CCL2), as well as reduced the expression of immunomodulatory M2 markers (CD206, IL4, IGF-1, TGF-β1, TGF-β2, TGF-β3, G-CSF and GM-CSF). Rosiglitazone could significantly decrease the mRNA expression of COX-2, iNOS, IFN-γ and TNF-α as shown in Fig. S1. However, pro-inflammatory cytokines (M1 markers) CD86, IL-1β, IL-6 and CCL2 and all anti-inflammatory cytokines (M2 markers) were not changed by PPARγ agonist rosiglitazone. Our results reveal that PPARγ activation could partly suppress LPS-induced M1 activation but did not promote a resting M2 microglial phenotype.

**
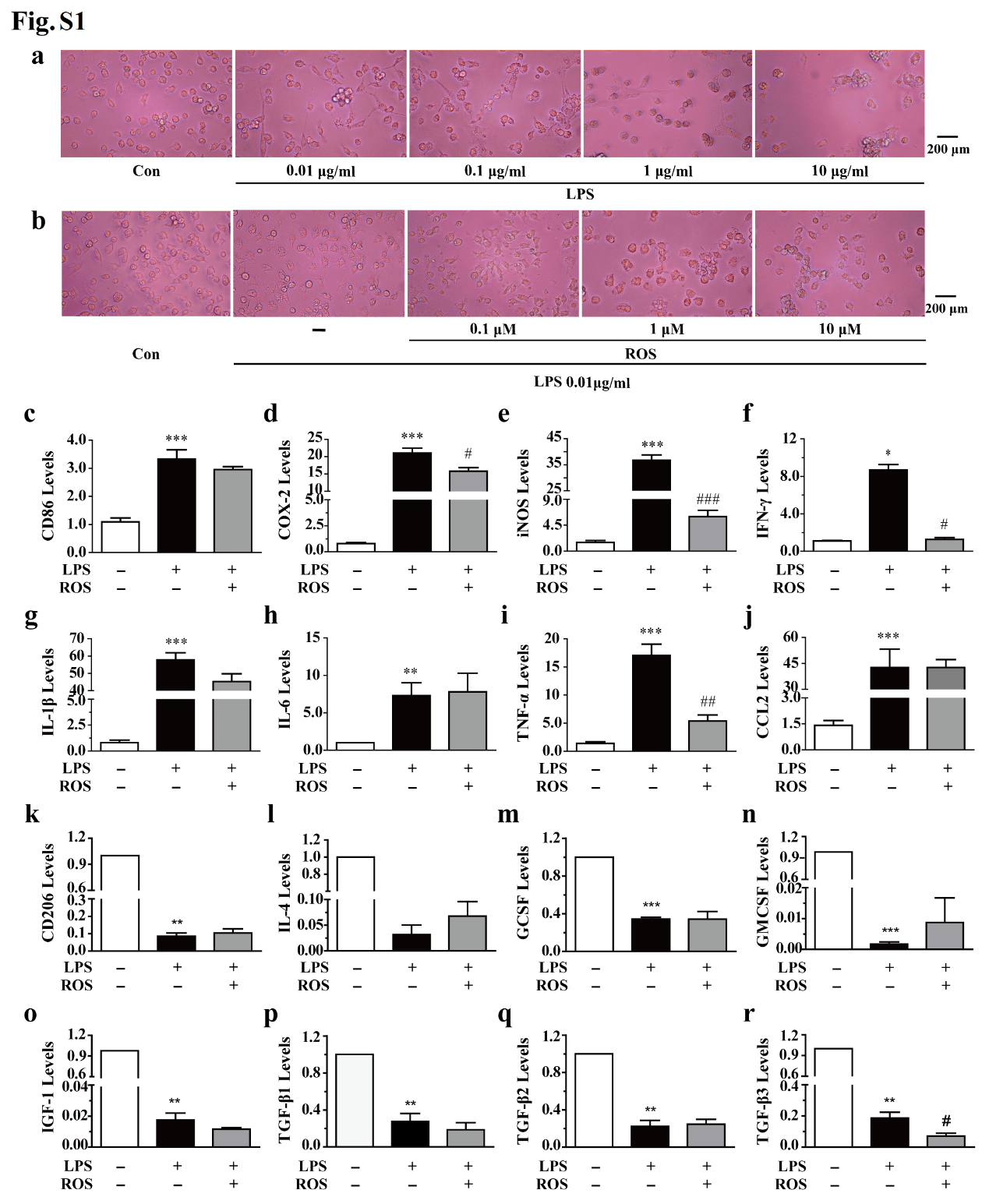
**

**Fig.S1 PPARγ activation could partly suppress LPS-induced M1 activation but did not promote a resting M2 microglial phenotype.** The morphology of microglial cells was observed by microscope. PPARγ agonistrosiglitazone did not influence the amoeboid “activated” morphology of microglia induced by LPS (a, b). Rosiglitazone signiﬁcantly inhibited LPS-induced mRNA expression of pro-inﬂammatory cytokines (TNF-a, COX-2,iNOS and IL-6) and anti-inﬂammatory factors (TGF-β3) in primary microglial cells (d-f, i, r), but did not alter the mRNA expression of M1and M2 marker, including CD86, IL-1β, IL-6, CCL2, CD206, IL-4, GCSF, GMCSF, IGF-1, TGF-β1 and TGF-β2 (c, g-h, j, k-q). Statistical analysis was performed using One-way ANOVA followed by Bonferroni’s post hoc test. Data are presented as means ± SEMs, n≧4, ^*^p < 0.05, ^**^p < 0.01, ^***^p < 0.001, compared with Control group;^#^p < 0.05, ^##^p < 0.01, ^###^p < 0.001, compared with LPS group.

**T0070907 does not alter the expressions of PPARα, PPARβ/δ and PPARγ in microglia**

As shown in Fig.S2, the protein expressions of PPARα, PPARβ/δ and PPARγ in the primary cultured microglia were determined by western blotting. The results showed that LPS treatment alone and LPS plus T070907 did not affect the expressions of PPARs.


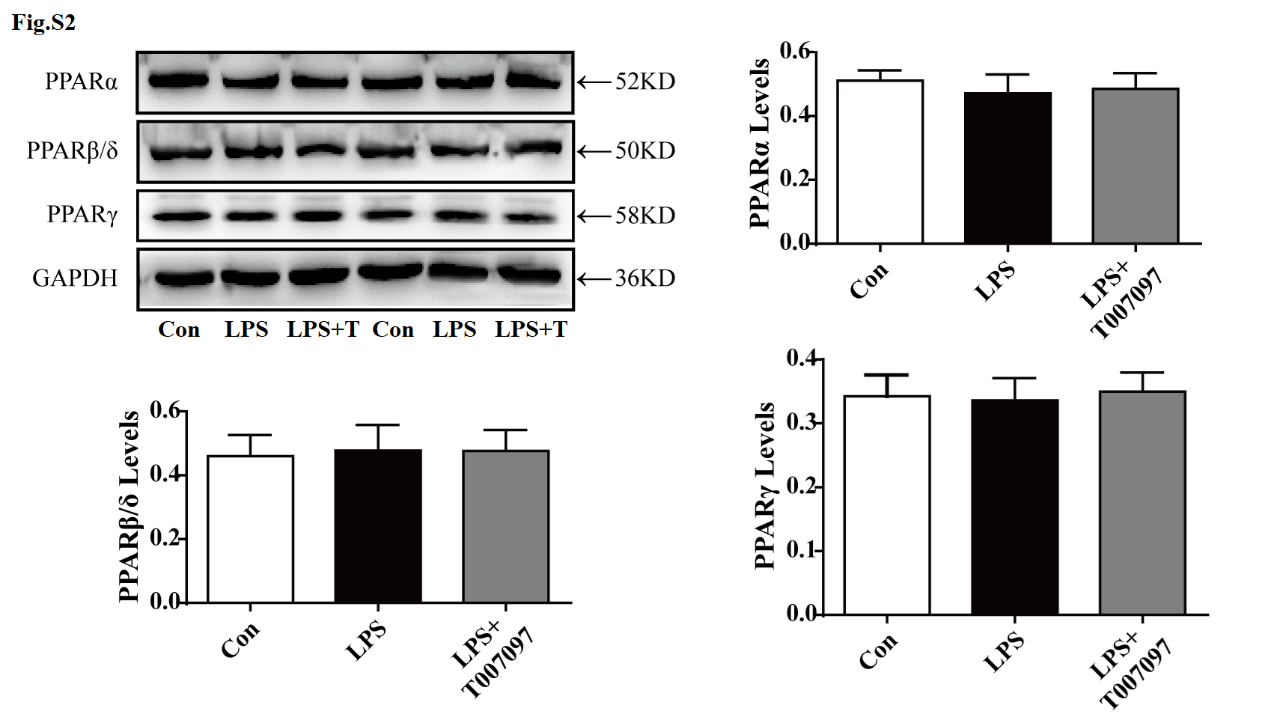


**Fig.S2. The effects of T0070907 on the expressions of PPARα, PPARβ/δ and PPARγ in microglia.** Data are presented as means ± SEMs, n≧4; Con: control; T: T0070907.
